# Optimized isolation and expansion of human airway epithelial basal cells from endobronchial biopsy samples

**DOI:** 10.1002/term.2466

**Published:** 2017-08-22

**Authors:** Kate H.C. Gowers, Robert E. Hynds, Ricky M. Thakrar, Bernadette Carroll, Martin A. Birchall, Sam M. Janes

**Affiliations:** ^1^ Lungs for Living Research Centre, UCL Respiratory University College London London UK; ^2^ Department of Thoracic Medicine University College Hospital London UK; ^3^ The Royal National Throat Nose and Ear Hospital UCL Ear Institute London UK

**Keywords:** adult stem cells, bioengineering, epithelial cells, primary cell culture, tissue transplantation, trachea

## Abstract

Autologous airway epithelial cells have been used in clinical tissue‐engineered airway transplantation procedures with a view to assisting mucosal regeneration and restoring mucociliary escalator function. However, limited time is available for epithelial cell expansion due to the urgent nature of these interventions and slow epithelial regeneration has been observed in patients. Human airway epithelial cells can be expanded from small biopsies or brushings taken during bronchoscopy procedures, but the optimal mode of tissue acquisition from patients has not been investigated. Here, we compared endobronchial brushing and endobronchial biopsy samples in terms of their cell number and their ability to initiate basal epithelial stem cell cultures. We found that direct co‐culture of samples with 3T3‐J2 feeder cells in culture medium containing a Rho‐associated protein kinase inhibitor, Y‐27632, led to the selective expansion of greater numbers of basal epithelial stem cells during the critical early stages of culture than traditional techniques. Additionally, we established the benefit of initiating cell cultures from cell suspensions, either using brushing samples or through enzymatic digestion of biopsies, over explant culture. Primary epithelial cell cultures were initiated from endobronchial biopsy samples that had been cryopreserved before the initiation of cell cultures, suggesting that cryopreservation could eliminate the requirement for close proximity between the clinical facility in which biopsy samples are taken and the specialist laboratory in which epithelial cells are cultured. Overall, our results suggest ways to expedite epithelial cell preparation in future airway cell therapy or bioengineered airway transplantation procedures.

## 1 INTRODUCTION

Airway tissue engineering has seen the development of cell‐scaffold solutions for otherwise intractable human disease and has seen clinical translation in compassionate cases (Badylak, Weiss, Caplan, & Macchiarini, 2012). Preclinical (Crowley, Birchall, & Seifalian, 2015) and early clinical work (Hamilton et al., 2015) suggests the importance of epithelial restoration following airway transplantation to avoid complications, such as recurrent infection (Zhang, Fu, & Xu, 2015). Clinical protocols have recognized the importance of epithelial replacement as bioengineered airway scaffolds have included cultured epithelial cells or explanted airway mucosa from recipient airways (Elliott et al., 2012). However, a standardized method to expand these cells is lacking, largely due to the varied clinical scenarios in which transplantation is indicated. Such technology would also be invaluable to inform the development of ‘standalone’ respiratory mucosal epithelial replacement treatments for a range of diseases. We and others have recently characterized an improved culture methodology (Chapman, Liu, Meyers, Schlegel, & McBride, 2010; Liu et al., 2012; Suprynowicz et al., 2012) for human airway basal cells, the stem/progenitor cells of the human upper airways (Hogan et al., 2014), by co‐culturing epithelial cells with mitotically inactivated 3T3‐J2 feeder cells in medium containing the Rho‐associated protein kinase inhibitor Y‐27632 (3T3+Y) (Butler et al., 2016; Reynolds et al., 2016). This method has clear advantages over the time‐consuming derivation of airway epithelial cells from pluripotent stem cells and conventional cell culture using bronchial epithelial growth medium (BEGM) for basal cell expansion, owing to its capability to expand autologous primary cells from living patients in meaningful numbers. However, the optimal method to isolate autologous epithelial cells from patient biopsy samples remains unclear. Previously, this has been achieved by explant culture of endobronchial biopsy samples in BEGM (Butler et al., 2016), but we reasoned that initiation of cultures would be improved using the 3T3+Y protocol.

**Figure 1:**
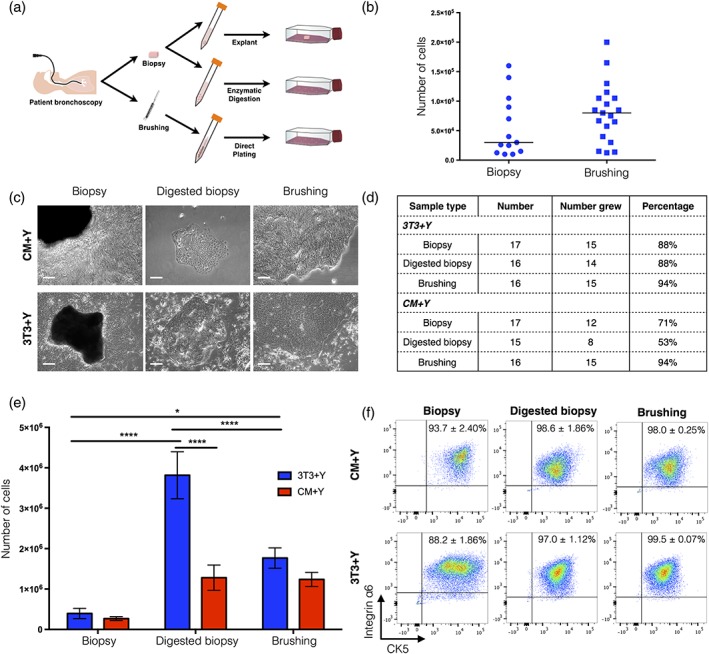
Comparison of cell outgrowth from endobronchial biopsies and brush biopsies. (a) Schematic representation of alternative methods to derive primary human airway epithelial cells from living donors. (b) Total number of cells contained within endobronchial biopsies and brushings. Each point represents one biopsy sample from five donors (biopsy) or 15 donors (brushing). (c) Brightfield images showing cell outgrowth from endobronchial biopsies, cell suspensions produced by dispase/trypsin digestion of endobronchial biopsies or from endobronchial brushings in either 3T3+Y co‐culture or 3T3‐J2‐conditioned medium + Y‐27632 (CM+Y). Scale bars indicate 100 μm. (d) Comparison of success rates of cell outgrowth. (e) Cell counts after trypsinization of epithelial outgrowths at day 12 of culture. A statistical analysis was performed using a two‐way ANOVA with Bonferroni post‐test; *p < 0.05; ****p < 0.0001; *n* = 8–14 biopsy samples within each condition (with a minimum of four donors sampled per group). (f) Flow cytometric analysis of cell outgrowths for basal epithelial cell markers cytokeratin 5 (CK5) and integrin α6. The percentage represents the mean ± standard error of the mean; *n* = 3–7 biopsy samples within each condition (with a minimum of two donors sampled per group)

*In vitro* expansion of human airway epithelial cells has been reported from both endobronchial brushings (Kelsen, Mardini, Zhou, Benovic, & Higgins, 1992) and endobronchial biopsies, either as explants (de Jong, van Sterkenburg, Kempenaar, Dijkman, & Ponec, 1993) or digested to obtain a cell suspension (Goulet et al., 1996). Initially we sought to compare two alternative modes of tissue acquisition to derive autologous airway cells from patients: endobronchial biopsy and endobronchial brushing samples (Figure 1a). Individuals consented to either endobronchial biopsies or endobronchial brushings, so we compared these modes of acquisition at the population level. We found a trend towards there being fewer cells in biopsy samples than in brushings, although this difference was not statistically significant (Figure 1b). This could be explained by the inefficiency of the digestion protocol used to isolate cells from biopsy tissue.

Next, we investigated methods to derive epithelial cell cultures from these endobronchial samples. We compared the expansion of epithelial cells from brushings and biopsies seeded directly in co‐culture as well as biopsies that were digested to a single cell suspension prior to seeding (Figure 1c). Cells were seeded both in direct 3T3+Y co‐culture and in medium conditioned by 3T3‐J2 feeder cells (CM+Y), as it has been previously reported that secreted factors mediate the effects of co‐culture and that direct contact with feeder cells is not required (Palechor‐Ceron et al., 2013). Cultures could be initiated from all sample types in both 3T3+Y and CM+Y, although the rate of successful initiation of cultures was higher in those expanded in 3T3+Y for biopsy and digested biopsy samples (Figure 1d). We found that explant biopsies generated the fewest cells by day 12 of culture, whereas digestion of biopsies to generate a single cell suspension prior to culture generated the greatest number of cells in 3T3+Y (Figure 1e). Cultures derived from brushings generated an intermediate number of epithelial cells at this time point in 3T3+Y, suggesting that, although brushing does generate a cell suspension, there might be fewer basal epithelial cells present in these samples. Digested biopsies expanded in CM+Y generated significantly fewer cells than those expanded in 3T3+Y, but for biopsy explants and brushings both 3T3+Y and CM+Y generated similar numbers of cells at the time of first passage (Figure 1e). Importantly, flow cytometric analyses suggested that, regardless of derivation or culture technique, cytokeratin 5 (CK5)‐/integrin α6‐expressing basal cells were selectively expanded from patient samples (Figure 1f).

**Figure 2:**
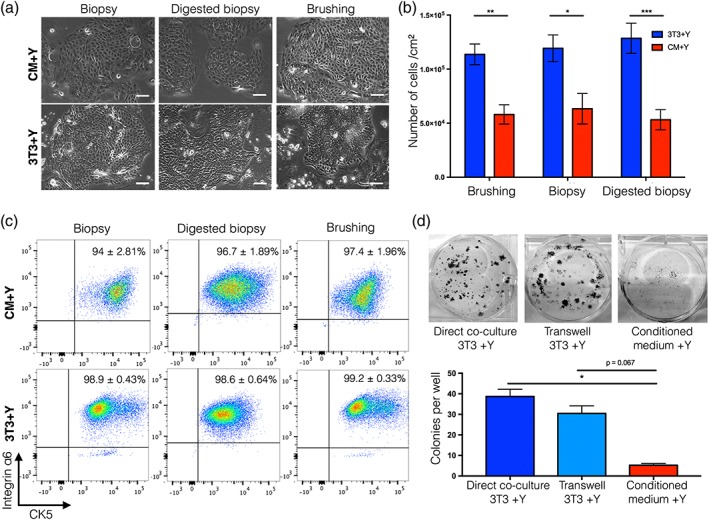
Comparison of cell expansion from endobronchial biopsies and brush biopsies after passage. (a) Brightfield images showing epithelial cell morphology of cultures derived from endobronchial biopsies, cell suspensions produced by dispase/trypsin digestion of endobronchial biopsies or from endobronchial brushings in either 3T3+Y co‐culture or 3T3‐J2‐conditioned medium + Y‐27632 (CM+Y) after passage. Scale bars indicate 50 μm. (b) Cell counts after trypsinization of epithelial outgrowths after 7 days of subculture. A statistical analysis was performed using a two‐way ANOVA with Bonferroni post‐test; *p < 0.05; **p = 0.01, ***p < 0.001; *n* = 7–13 donor cell cultures within each condition (with a minimum of four donors sampled per group). (c) Flow cytometric analysis of passage one epithelial cells for basal epithelial cell markers cytokeratin 5 (CK5) and integrin α6. The percentage represents the mean ± standard error of the mean; *n* = 3–9 biopsy samples from between two and four donors. (d) Representative images and quantification of colony‐forming assays to investigate the nature of 3T3‐J2 feeder cell support of human airway epithelial cells. Epithelial cells were grown in direct co‐culture with 3T3‐J2 cells, in indirect co‐culture with 3T3‐J2 cells (separated by a transwell) or in 3T3‐J2‐conditioned medium. A statistical analysis was performed using a Kruskal–Wallis test; *p < 0.05; colony‐forming assays were performed in triplicate using cells derived from three donors

After trypsinization and re‐seeding (that is, at ‘passage 1’), we found that basal cells derived from brushings and biopsies behaved similarly (Figure 2a), with comparable numbers generated in either 3T3+Y co‐culture or in CM+Y, although consistently fewer cells were expanded over the 7‐day culture period in CM+Y (Figure 2b). Again, CK5/integrin α6 co‐expression was demonstrated by flow cytometry (Figure 2c).

Next, we used low‐density colony formation assays to investigate the cause of the decreased numbers of basal cells expanded in CM+Y. Fewer epithelial colonies were generated in CM+Y than in direct 3T3+Y co‐culture but separation of epithelial cells from feeder cells by a transwell membrane allowed colony formation that was comparable with direct co‐culture (Figure 2d). This indicated that, although direct cell–cell contact between epithelial cells and feeder cells was dispensable for the enhancement of epithelial cell expansion, continuous production of secreted factors by feeder cells was important for epithelial support and could not be reproduced by feeding cells with 3T3‐J2‐conditioned medium three times per week.

**Figure 3:**
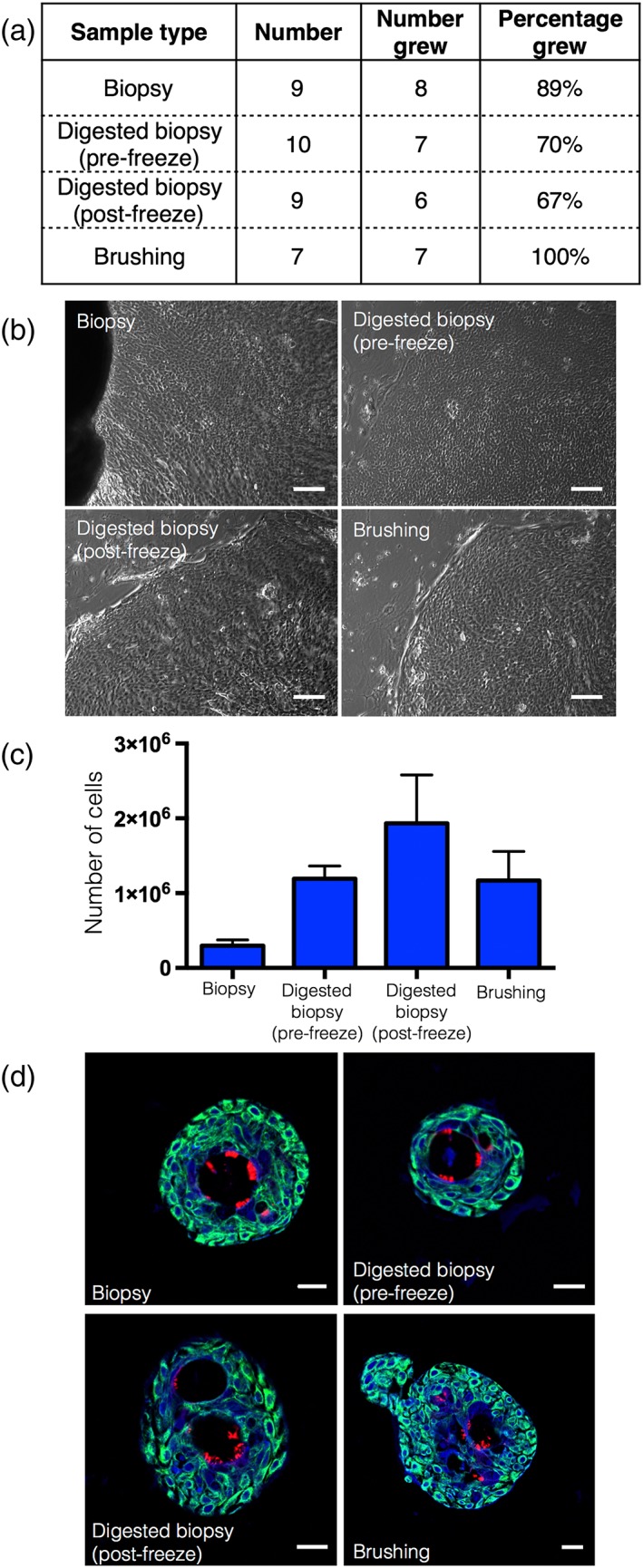
Cryopreservation of endobronchial biopsies allows subsequent derivation of epithelial cell cultures. (a) Comparison of success rates of epithelial cell outgrowth following cryopreservation. (b) Brightfield images showing epithelial cell outgrowths. Scale bars indicate 100 μm. (c) Cell counts after trypsinization of epithelial outgrowths from cryopreserved endobronchial biopsy/brushing samples after 14–17 days of culture [*n* = 6–8 biopsy samples within each condition (with a minimum of four donors sampled per group)]. (d) Immunofluorescence staining showing the presence of basal cells (cytokeratin 5; green) and multiciliated cells (acetylated α‐tubulin; red) in tracheospheres derived from a biopsy (top left), a biopsy digested before cryopreservation (top right), a biopsy digested after cryopreservation (bottom left) and a brushing (bottom right). DAPI (blue) was used as a counterstain. Scale bars indicate 20 μm

Finally, as organoid derivation was recently reported from cryopreserved primary tumours (Walsh, Cook, Sanders, Arteaga, & Skala, 2016), we investigated whether airway epithelial cell cultures could be initiated from cryopreserved patient samples. We generated cell cultures from cryopreserved samples with an efficiency of 89% for explant biopsies, 70% for biopsies digested prior to cryopreservation, 67% for biopsies digested after cryopreservation and 100% for brushings (Figure 3a, b). Again, we found that plating a single cell suspension, either by digesting a biopsy before or after cryopreservation or by using a brush biopsy, generated the highest number of cells (Figure 3c). To ensure that cryopreserved biopsy‐derived basal cells maintained their capacity for ciliated differentiation, we expanded cells for one further passage and used a three‐dimensional tracheosphere assay (Danahay et al., 2015) (Figure 3d).

To conclude, direct explant expansion of human airway epithelial cells in 3T3+Y culture conditions improves the early stages of culture compared with BEGM (88% culture success, approximately 400 000 cells after 12 days vs. 50% culture success, <250 000 cells after 2 weeks; Butler et al., 2016). However, the cell yield of explant biopsies is inefficient compared with initiating cultures from a cell suspension, either by digestion of biopsy samples (88% culture success, approximately 4 × 10^6^ cells after 12 days) or by collecting cells by brush biopsy (94% culture success, approximately 1.8 × 10^6^ cells after 12 days). We estimate that isolation of cells from a cell suspension in 3T3+Y could reduce the time required for epithelial cell expansion to 3–4 weeks in a case where only basal cells were to be transplanted on an adult tracheal scaffold. Colony formation assays suggested that airway epithelial cells required a continual supply of feeder products that was not recreated by feeding three times per week with 3T3‐J2‐conditioned medium: when cell–cell contact was prevented by adding 3T3‐J2 feeder cells on a transwell membrane, epithelial colony growth was comparable with growth in direct co‐culture. Notably, our study revealed that patient tissue can be cryopreserved prior to the initiation of cell cultures. Although cryopreservation did slow the initiation of epithelial cell cultures, this finding could remove the necessity for specialist clinical and research facilities to be in close proximity in future airway and mucosal tissue‐engineering clinical procedures. Current protocols limit the number of sites capable of performing autologous airway cell therapies to those with both appropriate clinical and research facilities, but samples could be cryopreserved for transportation, expanding the potential application of these techniques. Overall, our findings suggest methods to facilitate cell preparation in future bioengineered airway transplantation procedures.

## CONFLICT OF INTEREST

The authors declare no conflicts of interest.

## Supporting information

Data S1. Supplementary Methods
The following supporting information may be found in the online version of this article: Click here for additional data file.
