# Supplementary material for: Optimized isolation and expansion of human airway epithelial basal cells from endobronchial biopsy samples
Source: J Tissue Eng Regen Med. 2017 Aug 22;12(1):e313–7. doi: 10.1002/term.2466 (PMC5811901; doi:10.1002/term.2466)
Supplement: Supplementary file 1 — Data S1. Supplementary Methods The following supporting information may be found in the online version of this article: [file TERM-12-e313-s001.docx]

**Optimized isolation and expansion of human airway epithelial basal cells from endobronchial biopsy samples**

Kate H. C. Gowers (1), Robert E. Hynds (1), Ricky M. Thakrar (1,2), Bernadette Carroll (1,2), Martin A. Birchall (3) and Sam M. Janes (1)

1) Lungs for Living Research Centre, UCL Respiratory, University College London, London, UK

2) Department of Thoracic Medicine, University College Hospital, London, UK

3) UCL Ear Institute, The Royal National Throat Nose and Ear Hospital, London, UK

**Supplementary Methods**

**Human tissue**

Primary human airway samples were obtained from healthy airway regions during tracheobronchoscopy procedures with patient consent and ethical approval (UK research ethics committee reference 06/Q0505/12). Endobronchial biopsy samples were transported to the laboratory in a transport medium consisting of αMEM supplemented with 1X penicillin/streptomycin (Gibco; 15070) and 250ng/ml amphotericin B (Fisher Scientific; 10746254).

**Enzymatic digestion of endobronchial biopsy samples**

Where indicated, endobronchial biopsy samples were digested using 16 U/ml dispase (Corning) in RPMI (Gibco) for 20 minutes at room temperature. Epithelium was dissected away and fetal bovine serum (FBS) was added to the dispase solution to a final concentration of 10% FBS and set aside. Both epithelial and non-epithelial components were digested in 0.1% trypsin/EDTA (Sigma) at 37°C for 30 minutes with agitation by pipetting every 10 minutes (Hegab *et al.*, 2012). Digests were neutralized with FBS to a final concentration of 10% and combined with the neutralized dispase solution. Cells were centrifuged and resuspended in culture medium for counting. Viable cell number was determined using trypan blue and a haemocytometer.

**Feeder cell culture**

3T3-J2 mouse embryonic fibroblasts were cultured in Dulbecco’s modified Eagle’s medium (DMEM; Gibco #41966) supplemented with 100 U/ml penicillin, 100 µg/ml streptomycin (Gibco; #15070) and 9% bovine serum (Gibco; #26170). Cells were cultured at 37**°**C with 5% CO_2_ with three changes of medium per week. To generate feeder layers, cells were mitotically inactivated by treatment with 4 µg/ml mitomycin C (Sigma; M4287) in culture medium for 2 hours. Cells were trypsinized and plated at a density of 2 x 10^4^ cells/cm^2^ in growth medium. Epithelial cells were added in epithelial culture medium the following day.

**Human airway epithelial cell culture**

3T3+Y culture conditions for human airway epithelial cells were as previously described (Butler *et al.*, 2016; Hynds *et al.*, 2016). Epithelial culture medium consisted of DMEM (Gibco; 41966) and F12 (Gibco; 21765) in a 3:1 ratio with penicillin/streptomycin (Gibco; 15070) and 5% FBS (Gibco; 10270) supplemented with 5 μM Y-27632 (Cambridge Bioscience; Y1000), 25 ng/ml hydrocortisone (Sigma; H0888), 0.125 ng/ml EGF (Sino Biological; 10605), 5 μg/ml insulin (Sigma; I6634), 0.1 nM cholera toxin (Sigma; C8052), 250 ng/ml amphotericin B (Fisher Scientific; 10746254) and 10 μg/ml gentamycin (Gibco; 15710). Cells were maintained at 37**°**C with 5% CO_2_ and medium was changed three times per week. To separate epithelial cells from feeder cells we used differential trypsinization. Here, trypsin was applied to co-cultures for 60 seconds at 37**°**C, after which time the feeder cells, but not the strongly adherent epithelial cells, were removed. Flasks were washed with PBS and the remaining epithelial cells were then re-trypsinized. Viable cell number was determined using trypan blue and a haemocytometer.

**Colony-forming assays**

Colony formation was assessed by seeding 1000 primary human airway epithelial cells in pre-coated collagen I-coated 6-well plates (rat tail collagen I; BD #354236). 3T3-J2 feeder cells were seeded at 2 x 10^4^ cells/cm^2^ the day before epithelial cell seeding. Cells were fed three times a week and fixed and stained with 1% crystal violet solution (Sigma) after 10 days. Plates were washed extensively in water and allowed to dry at room temperature overnight. Colonies of more than 10 cells were counted using a brightfield microscope.

**3D tracheospheres**

Tracheospheres were generated as previously described (Butler *et al.*, 2016; Hynds *et al.*, 2016). Basal epithelial cells cultured in 3T3+Y were separated from feeder cells by differential trypsinization. Ultra-low attachment 96-well plates (Corning; clear, flat bottom) were lined with 30 μl 25% Matrigel (in tracheosphere medium; BD Biosciences; growth factor reduced) and allowed to gel at 37^o^C for 20 minutes. Tracheosphere medium was 50% bronchial epithelial basal medium (Lonza) and 50% DMEM (Gibco; #41966). BEGM supplements (Lonza) (excluding triiodothyronine, gentamycin, amphotericin and retinoic acid) were added. Medium was supplemented with 100 nM retinoic acid (Sigma) immediately before each use. 2,500 basal cells were seeded per well in 65 μl 5% Matrigel (in tracheosphere medium; BD Biosciences; growth factor reduced). Cells were fed by addition of 70 μl tracheosphere medium on day 3, day 8 and day 14. On day 18, tracheospheres were collected by centrifugation (200 *g* for 3 minutes) in cold PBS. Tracheospheres were fixed by resuspension in 4% PFA for 30 minutes, washed once with PBS and resuspended in Histogel specimen-processing gel (Thermo Fisher) for processing and paraffin embedding. 5 µm sections were stained for immunofluorescence: slides were dewaxed using an automated staining system (Tissue-Tek) and antigen retrieval was performed using citrate buffer. Slides were then blocked in PBS containing 10% FBS and stained in this blocking buffer overnight at 4**°**C with an anti-acetylated α-tubulin (ACT) antibody (Sigma; T6793) and an anti-cytokeratin 5 (CK5) antibody (Abcam; ab52635). After washing in PBS, species-appropriate secondary antibodies (1:500; AlexaFluor dyes; Molecular Probes) were applied in blocking buffer at room temperature for 2 hours. DAPI was added as a counterstain. Images were acquired using a Zeiss LSM700 confocal microscope.

**3T3-J2 fibroblast feeder cell-conditioned medium**

Conditioned medium was generated by plating mitotically inactivated 3T3-J2s at feeder cell density and adding epithelial growth medium (without Y-27632) 24 hours later. Medium was collected and changed after 24 and 48 hours, before being combined and filtered. Fresh epithelial growth medium without Y-27632 was added to the conditioned medium at a ratio of 3 parts conditioned medium to 1 part fresh medium and the combined medium was supplemented with Y-27632 at a concentration of 5 μM. Medium was aliquoted and stored at -80°C until needed.

**Cryopreservation**

For cryopreservation of samples prior to culture, samples were resuspended in a solution of 50% epithelial growth medium + Y-27632, 42.5% Profreeze (Lonza) and 7.5% DMSO. Samples were frozen slowly to -80°C in Mr. Frosty freezing containers before being transferred to liquid nitrogen for long-term storage.

**Flow cytometry**

After initial outgrowth (P0) and after expansion at passage 1 (P1), epithelial cells were trypsinized to generate a single cell suspension. For epithelial cells expanded in 3T3+Y, differential trypsinization was performed. 200,000 cells were blocked in 10% FBS in FACS buffer (PBS containing 1% bovine serum albumin (BSA)) for 20 minutes at 4^o^C before being stained with PE-conjugated integrin α6 (BD Biosciences; 1:20) in FACS buffer for a further 20 minutes at 4^o^C. For intracellular staining, cells were fixed with BD Cytofix Fixation Buffer (BD Biosciences) at 4^o^C for 15 minutes. Cells were incubated with APC-conjugated anti-cytokeratin 5 (Abcam) in permeabilization buffer (eBioscience) for 20 minutes at 4^o^C. Prior to staining, the anti-cytokeratin 5 antibody was conjugated to APC using a Zenon labeling kit (Thermo Fisher) according to the manufacturer’s instructions. 1 μg/ml DAPI was added to ensure only living cells were analyzed. Flow cytometry was performed using an LSRFortessa (BD Biosciences) and analyzed using FlowJo 10.0.6 (Tree Star).

**Statistical analysis**

Statistical analyses were carried out using GraphPad Prism as indicated in the figure legends.

**Supplementary references**

Butler CR, Hynds RE, Gowers KH*, et al.* 2016; Rapid expansion of human epithelial stem cells suitable for airway tissue engineering. *Am J Respir Crit Care Med*.

Hegab AE, Ha VL, Attiga YS*, et al.* 2012; Isolation of basal cells and submucosal gland duct cells from mouse trachea. *J Vis Exp*: e3731.

Hynds RE, Butler CR, Janes SM*, et al.* 2016; Expansion of human airway basal stem cells and their differentiation as 3d tracheospheres. *Methods Mol Biol*.
